# Biotin conjugates in targeted drug delivery: is it mediated by a biotin transporter, a yet to be identified receptor, or (an)other unknown mechanism(s)?

**DOI:** 10.1080/14756366.2023.2276663

**Published:** 2023-11-13

**Authors:** Ravi Tripathi, Anchala Guglani, Rujuta Ghorpade, Binghe Wang

**Affiliations:** a Department of Chemistry and Center for Diagnostics and Therapeutics, Georgia State University, Atlanta, GA, USA; b Department of Biology, Georgia State University, Atlanta, GA, USA

**Keywords:** Biotin, targeted drug delivery, SMVT, biotin receptor, SAR

## Abstract

Conjugation of drugs with biotin is a widely studied strategy for targeted drug delivery. The structure–activity relationship (SAR) studies through H^3^-biotin competition experiments conclude with the presence of a free carboxylic acid being essential for its uptake via the sodium-dependent multivitamin transporter (SMVT, the major biotin transporter). However, biotin conjugation with a payload requires modification of the carboxylic acid to an amide or ester group. Then, there is the question as to how/whether the uptake of biotin conjugates goes through the SMVT. If not, then what is the mechanism? Herein, we present known uptake mechanisms of biotin and its applications reported in the literature. We also critically analyse possible uptake mechanism(s) of biotin conjugates to address the disconnect between the results from SMVT-based SAR and “biotin-facilitated” targeted drug delivery. We believe understanding the uptake mechanism of biotin conjugates is critical for their future applications and further development.

## Graphical abstract

**Figure:**
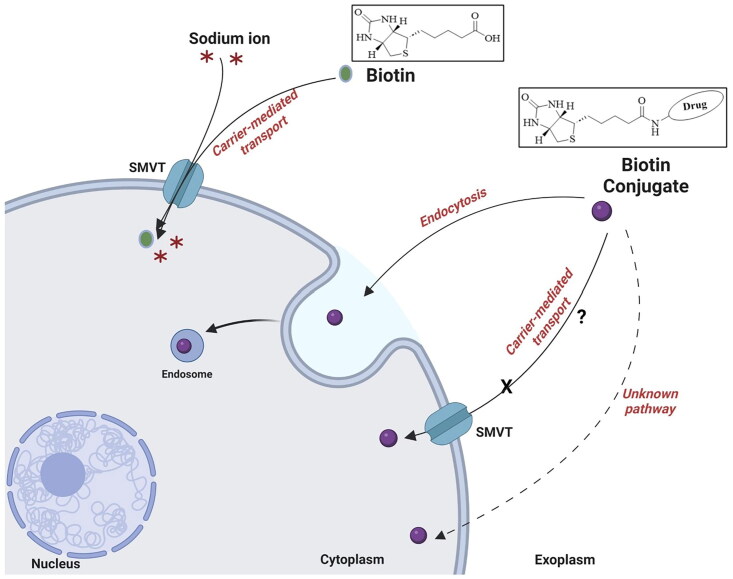

## Introduction

Even with the tremendous development in the area of drug discovery and development during the last 50 years^1^, there is still a large number of diseases that do not have effective treatment^2^. One can safely say that the levels of challenges are becoming progressively higher in dealing with the remaining problems, even after taking into consideration of new technologies and new scientific discoveries/insights. Future work is almost entirely focused on targeted therapies of various types, including modulation of activities of the molecular target(s) implicated in the relevant pathologies and/or targeted delivery based on molecular biomarkers. The latter often involves conjugation of a therapeutic molecule with a targeting ligand such as peptides^3–5^, nucleic acids^6^^,^^7^, micronutrients^8–11^, and antibodies^12^^,^^13^ as well as the use of nanomaterials^14^^,^^15^ to achieve the goals of targeted delivery. Such targeting ligands act as vectors and offer distinct physicochemical^16–19^ and pharmacological^8^^,^^20–22^ features resulting in improved pharmacodynamic and pharmacokinetic profiles of the appended drugs^2^. We have a long-standing interest in developing novel drug delivery approaches including targeted drug delivery^17^^,^^23–30^. Along this line, we have an interest in biotin-mediated targeted delivery of drugs to organs such as the kidney, colon, and the lung as well as cancer^31–33^.

Biotin^34^ is a cofactor for enzymes collectively referred to as "biotin-dependent carboxylases," which are involved in the metabolism of amino acids, fatty acids, carbohydrates, and urea^35–37^. In addition, the roles of biotin also extend to the regulation of cellular processes, including gene expression and cell signalling^38^. Physiological concentrations of biotin have been reported to be in the low nanomolar range^39–44^. It has been reported that cancer cells overexpress biotin uptake system(s), which often leads to enhanced intracellular uptake of biotin^8^^,^^31^^,^^41^^,^^45^. A few examples are ovarian, lung, renal, colon, and breast cancers as well as leukaemia^8^^,^^38^^,^^46^. Conceivably, the over-expression of biotin uptake system(s) in a given type of cells provides a valuable strategy to selectively target the relevant cell types with a biotin analogue that are recognised by these “uptake system(s)”. The very first example of “biotin-mediated” drug delivery with the aim of targeting the biotin uptake system was published in 1990 describing work in plant cells (soybean)^47^. Along this line, it should be noted that earlier work using biotin–avidin/streptavidin interactions for drug delivery was not intended to target the biotin uptake system^48^^,^^49^. Interestingly, the very first paper on using biotin conjugates for drug delivery in mammalian cells was not for its ability to bind to the biotin uptake protein^50–58^. The very first mention of synthesising a biotin conjugate for taking advantage of the biotin uptake system in animals was reported in 2001 on the oral absorption properties of biotinylated retro-inverso Tat nonapeptides^59^. This study demonstrated an enhancement of up to 500-fold in transporting the biotinylated version of the peptide. Such transport was shown to be concentration dependent, saturable, and inhibitable by biotin. In 2004, a comparative study by Russell-Jones et al.^8^ examined the uptake of fluorescently labelled polymers conjugated with biotin, folic acid, and vitamin B_12_ in 18 different tumour cell lines *in vitro* and *in vivo*. Compared to the non-targeted polymer, an enhanced uptake of all the ligand-conjugated polymers was found in various cell lines. For instance, Colo-26 cells (murine colon tumour) treated with vitamin B_12_-conjugated and biotin-conjugated polymers showed >2-fold more fluorescence intensity than that of non-targeted polymers. Of note, compared to the folic acid-conjugated and vitamin B_12_-conjugated polymers, the uptake of biotin-conjugated polymers was found to be >3-fold higher in M109 cells (murine lung carcinoma). Since then, biotin has been extensively studied as a vector for targeted delivery of anticancer agents^31^^,^^46^^,^^60–64^. For information on biotin metabolism, biological functions, and its medical applications, readers are referred to several comprehensive reviews^31^^,^^32^^,^^34–36^^,^^45^^,^^65–78^.

**Figure 1.**
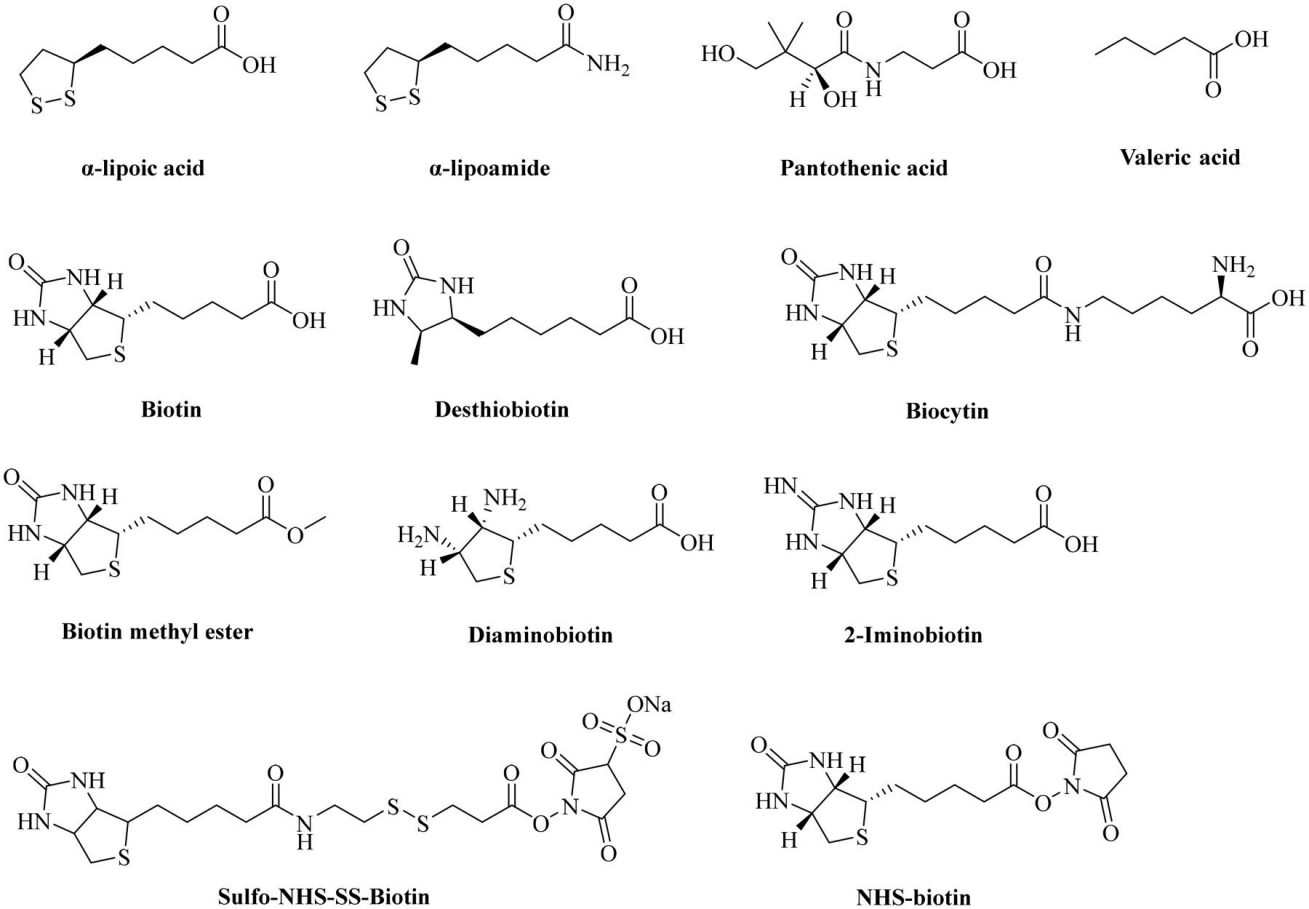
Chemical structures of α-lipoic acid, pantothenic acid, biotin, and its structural analogs.

While there has been very impressive progress in using biotin conjugates for targeted delivery applications in cell culture and animal models, much less is known about the mechanistic aspects of such success. Foremost, it is not clear whether such targeted delivery relies on a biotin transporter or receptor. Though literature discussions seem to use the terms biotin “receptor” and “transporter” without a clear distinction, these are indeed two distinctly different concepts^46^^,^^79–86^. It is commonly accepted that “transporters mediate the passage of molecules across cell membranes by alternating between inward- and outward-facing states, while receptors undergo intracellular structural rearrangements that initiate signaling cascades^87^.” Known biotin transporters almost universally require the presence of a free carboxylic acid group for the recognition of biotin (Figure 1)^33^^,^^72^^,^^82^^,^^88–92^, and yet the biotin conjugates used in targeted delivery transforms the carboxyl group to an amide or ester^32^^,^^45^, which is supposed to abolish the ability for a biotin transporter to recognise such a conjugate^72^. At the same time, no known “biotin receptor” has been identified, only transporters^72^^,^^73^^,^^88^^,^^91^^,^^93^. Further, there is a report of the “anti-inhibition” effects of biotin in the binding of a biotin conjugate with cells that express the biotin transporter^94^. In addition, a study by Wei and coworkers reported the lack of competition between 2000-fold excess of biotin and biotin conjugate during the uptake experiment in *E. coli*^95^. All these suggest the need for much more mechanistic studies on how biotin conjugation allows for “biotin-receptor/transporter”-mediated delivery. In this review, we provide a brief overview of the known biotin transporters, discuss known structure–activity relationships (SARs) in binding between a substrate and a biotin transporter, explore the disconnect between the SAR results and “biotin receptor/transporter-mediated" delivery of biotin conjugate, and suggest various possibilities. All these are aimed to help future design of biotin conjugates for efficient delivery with a firm understanding of the biology behind it. Below, we provide our critical analysis by starting with a description of the various known biotin transport systems.

## Known biotin uptake systems

**Figure 2.**
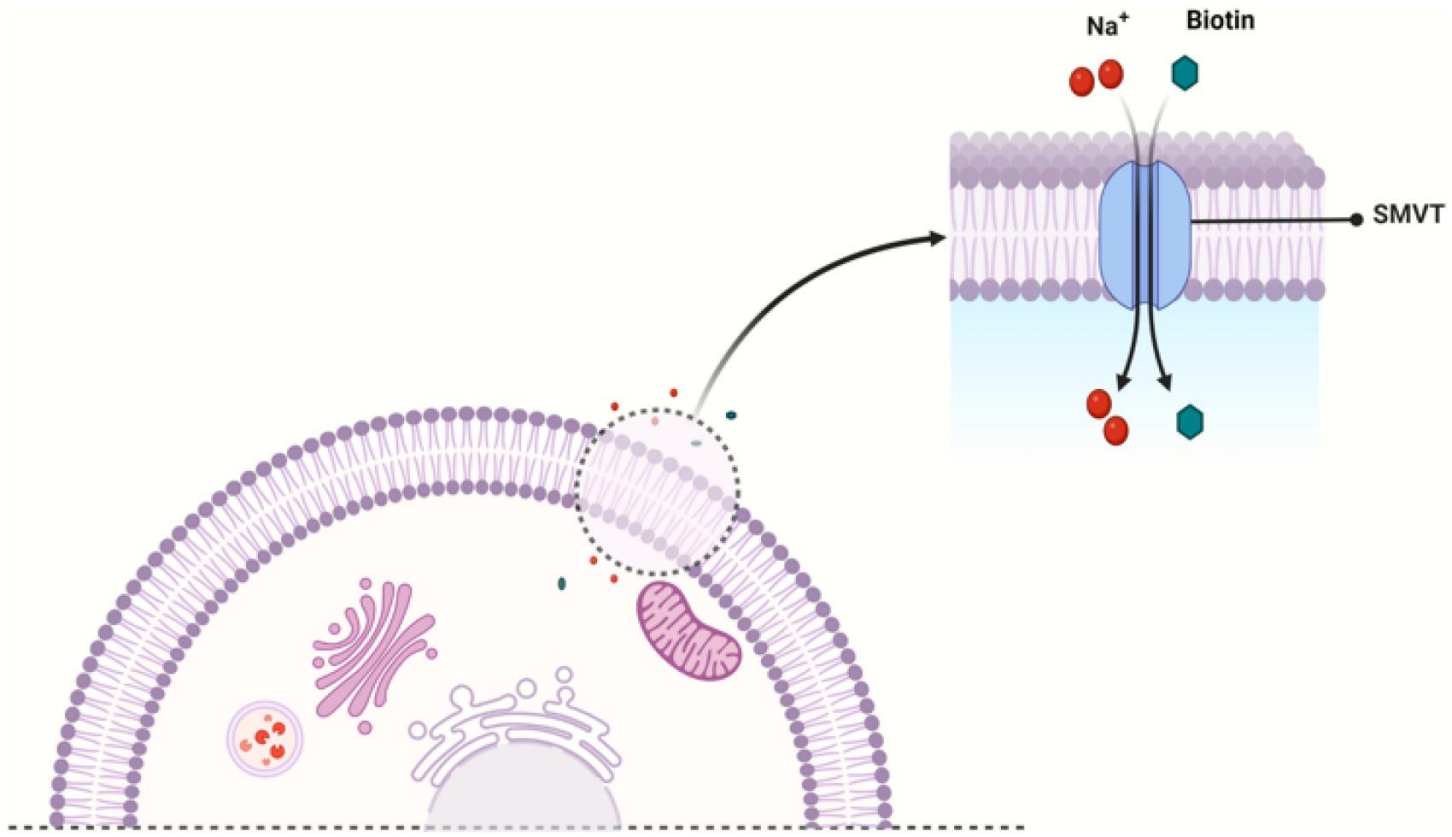
A proposed mechanism of SMVT-mediated uptake of biotin by a mammalian cell.

The uptake of biotin into cells occurs mainly via the sodium-dependent multivitamin transporter (SMVT)^96^, which employs a transmembrane Na^+^ gradient for its translocation (Figure 2)^32^. In addition, a few studies have revealed a carrier system other than SMVT that might mediate biotin uptake in human peripheral blood mononuclear cells (PBMC)^97^ and keratinocytes^98^. In the following subsections, we briefly discuss all the known biotin uptake systems in the mammalian cell.

### Sodium-dependent multivitamin transporter

SMVT, a product of the solute carrier family-5 member-6 (SLC5A6) gene, is responsible for the transportation of biotin, lipoic acid, and pantothenic acid (Figure 1) across the cell membrane^70^^,^^72^^,^^99^. This transport is coupled with Na^+^ flux^99^. As proposed based on topographical analysis^100^^,^^101^, the SMVT consists of 635 amino acids and has 12 putative transmembrane domains with *N*- and *C*-termini towards the cytosol. The nucleotide sequence of the cDNA predicts a molecular weight of 68.6 kDa^70^^,^^102^. It also has four *N*-linked glycosylation sites in extracellular domains and two protein kinase C-dependent phosphorylation sites in cytoplasmic domains^100^^,^^103^. SMVT distributes ubiquitously in the human body and is most abundantly expressed in the absorptive tissues of the liver, intestine, placenta, pancreas, and kidney^100^^,^^104^. Functionally, SMVT transports solutes such as biotin across the cell membrane against an electrochemical gradient of Na^+^. One biotin molecule is co-transported with two sodium ions in a single transport cycle (Figure 2)^100^. Again, SMVT has been widely studied for targeted delivery of biotin conjugates into cells overexpressing SMVT^105^; although, known SAR binding for SMVT does not support such delivery.

### Monocarboxylate transporter-1 (MCT-1)

The MCT family of proteins belongs to the proton-dependent transport protein family (SLC16A), with 14 members based on sequence homology^106^^,^^107^. These transporters mediate the uptake of short-chain monocarboxylates such as lactate, α-hydroxybutyrate, pyruvate, and biotin in mammalian cells^96^. Among these proton-dependent transporters, MCT-1 to MCT-4 mediate the uptake of endogenous monocarboxylates into the brain, kidney, and intestinal cells through the electroneutral co-transport of one monocarboxylate molecule with one proton (1:1)^108^. MCT-1 has been reported as an alternative biotin transporter in mammalian lymphoid cells; although, these cells also express SMVT^97^. MCT-1 utilises a sodium-dependent co-transport mechanism for uptake into the PBMCs^109^. The Michaelis–Menten constant (*K*_m_) was reported to be 2.6 nM, ∼1000-times lower for MCT-1-mediated biotin transport in PMBCs than SMVT-mediated biotin transport^97^^,^^109^. Of note, carrier-mediated transport exhibits Michaelis–Menten kinetics; hence, *K*_m_ is a widely used parameter to indicate the binding affinity (alternatively, specificity) of the substrates with the carrier proteins^110–113^.

### Miscellaneous: uptake of biotin into keratinocytes

The liver, intestinal epithelia, kidney, and placenta express SMVT for biotin uptake^97^. In addition, Grafe et al.^98^ studied the uptake of biotin in the human HaCaT cell line and native non-transformed keratinocytes and revealed that lipoic acid and pantothenic acid inhibit the Na^+^-dependent biotin uptake in keratinocytes. SMVT showed an apparent Michaelis–Menten constant of 23 µM for biotin, 1 µM for pantothenic acid, and 4.6 µM for lipoic acid (oxidised form). In addition, kinetic studies showed the presence of a second uptake system for biotin in the human keratinocytes with an apparent Michaelis–Menten constant of 2.6 nM. Interestingly, this value is same as that for the MCT-1-mediated biotin transport in PMBCs. This uptake system seems specific for biotin because the uptake was not inhibited by lipoic acid and pantothenic acid. In comparison, SMVT-mediated biotin uptake was significantly affected by both lipoic acid and pantothenic acid. Hence, the study concluded that human keratinocytes express both the SMVT and another biotin-specific carrier-mediated transport system.

## The structure–activity relationship: what might the SAR imply in terms of uptake mechanism(s)?

SMVT exhibits broad ligand specificity as it recognises a range of molecules such as biotin, pantothenic acid (vitamin B_5_), α-lipoic acid, and iodide for their cellular uptake^31^^,^^70^^,^^114^. Another transporter MCT-1 recognises short-chain aliphatic acids such as lactate, pyruvate, butyrate, acetoacetate, β-hydroxybutyrate, and γ-hydroxybutyrate and seems to be an even more versatile transporter than SMVT^96^^,^^106^. Because MCT-1 is largely involved in the transport of endogenous aliphatic carboxylates and has less prominent roles in biotin transport, it has not been as widely studied as the SMVT in terms of understanding the SAR for their binding characteristics. Therefore, we analyse the SAR of SMVT substrate/ligand characteristics as summarised in Table 1. The substrate specificity of SMVT has been studied by determining the uptake kinetics of radiolabeled biotin in the presence of unlabelled biotin and its structural analogues such as α-lipoic acid, α-lipoic amide (α-lipoamide), biocytin, desthiobiotin, biotin methyl ester, and pantothenic acid (Figure 1)^89^^,^^91^^,^^92^^,^^116^. For instance, Nylander and coworkers examined the uptake of biotin (5 µM) in the presence of its structural analogues in human intestinal brush border membrane (entry 1, Table 1)^91^. At 20 and 50 µM, desthiobiotin inhibited the uptake of biotin to 5.25 ± 0.18 pmol.mg protein^−1^.20 s^−1^ and 4.88 ± 0.14 pmol.mg protein^−1^.20 s^−1^, respectively, when compared to the control group (9.80 ± 0.60 pmol.mg protein^−1^.20 s^−1^). Similarly, α-lipoic acid also diminished the uptake to 5.60 ± 0.22 pmol.mg protein^−1^.20 s^−1^ and 4.70 ± 0.27 pmol.mg protein^−1^.20 s^−1^ at 20 and 50 µM concentrations, respectively in comparison to the control group. However, uptake studies using biotin analogues having modified carboxylic acid such as biotin methyl ester and α-lipoic amide did not result in a marked decrease in biotin uptake by intestinal cells. Another study done by Mitra and coworkers investigated the uptake of 10 nM [^3^H]biotin in a human-derived retinoblastoma cell line (Y-79) (entry 4, Table 1)^93^. In the presence of 1 mM of unlabelled biotin, valeric acid, pantothenic acid, α-lipoic acid, and desthiobiotin, the uptake of [^3^H]biotin decreased to 9.03 ± 1.51%, 60.39 ± 4.02%, 9.12 ± 1.39%, 6.97 ± 0.82%, and 15.61 ± 2.41% of the control group, respectively. Treatment with 1 mM of biocytin and biotin methyl ester did not produce any inhibitory effect on [^3^H]biotin uptake. Therefore, it is clear that SMVT does not interact with biotin analogues having modified carboxyl group. Further, Said and coworkers conducted a similar study in the human intestinal cell line Caco-2 (entry 2, Table 1)^88^. Specifically, incubation of cells with [^3^H]biotin (4 nM) with 25 µM of biotin and desthiobiotin resulted in the decrease of [^3^H]biotin uptake from 1.69 ± 0.04 pmol.mg protein^−1^.3 min^−1^ to 0.664 ± 0.03 and 0.95 ± 0.02 pmol.mg protein^−1^.3 min^−1^, respectively. However, no significant inhibitory effects on [^3^H]biotin uptake were observed upon incubation with 25 µM of diaminobiotin, biotin methyl ester, and biocytin. A few conclusions can be drawn. First, desthiobiotin (devoid of the tetrahydrothiophene ring present in biotin) shows strong inhibition of biotin uptake by SMVT (entries 1–5)^33^^,^^88^^,^^89^^,^^91^. Similarly, α-lipoic acid (devoid of the tetrahydroimidazolone ring present in biotin) competes strongly with biotin for SMVT-mediated transport (entries 1, 3, and 4)^33^^,^^91^^,^^93^. Such results indicate that the presence of two fused five-membered rings is not essential. Second, diaminobiotin with two ionisable (protonation) amino groups on a single five-membered ring does not compete strongly with biotin (entry 2, Table 1). In addition, the 2-iminobiotin compound (Figure 1) that contains fused rings do not compete with biotin for SMVT-mediated transport. Obviously, 2-iminobiotin has a protonatable guanidine moiety instead of a urea moiety in the second five-membered ring. Though the SAR for biotin interaction with the SMVT seems to be complex^59^^,^^88^^,^^100^, the presence of a protonatable functional group clearly is not favourable for such interactions. Third, valeric acid was also found to be a strong inhibitor of biotin transport by the SMVT although it does not contain fused tetrahydroimidazolone and tetrahydrothiophene rings in its structure (entries 3 and 4, Table 1)^33^^,^^93^. The presence of a free aliphatic carboxylic acid group is the only common feature that exists among these structurally diverse substrates of SMVT^117^. As expected, modification of the carboxylic acid group to an ester (e.g. biotin methyl ester) or amide (e.g. biocytin and α-lipoic amide) led to the abolishment of the ability to compete with biotin for transport by SMVT (entries 1, 2, and 5, Table 1)^88^^,^^89^^,^^91^^,^^118^.

**Table 1.** Uptake studies of biotin in various cell lines.

Entry	Cells	Substrates used in the competition assay		Effect on biotin transport by SMVT	Conclusion	Ref.
1	Human intestinal tissue (brush-border membrane vesicle)	Biotin	Desthiobiotin	Biotin (5 µM) uptake reduced from 9.80 ± 0.60 pmol.mg protein^–1^.20 s^–1^ (control group) to 5.25 ± 0.18 pmol.mg protein^–1^.20 s^–1^ and 4.88 ± 0.14 pmol.mg protein^–1^.20 s^–1^ in the presence of 20 and 50 µM of desthiobiotin, respectively.	These structural analogs of biotin reduce biotin transport by SMVT at 20–50 mM in the competition assay. The absence of tetrahydroimidazolone ring (α-lipoic acid) or tetrahydrothiophene (desthiobiotin) in the structure of biotin does not significantly alter their interaction with the SMVT.	91
	
			α-Lipoic acid	Biotin (5 µM) uptake reduced from 9.80 ± 0.60 pmol.mg protein^–1^.20 s^–1^ (control group) to be 5.60 ± 0.22 pmol.mg protein^–1^.20 s^–1^ and 4.70 ± 0.27 pmol.mg protein^–1^.20 s^–1^ in the presence of 20 and 50 µM of α-lipoic acid, respectively.		
			Biotin methyl ester	Biotin (5 µM) uptake reduced slightly from 9.80 ± 0.60 pmol.mg protein^–1^.20 s^–1^ (control group) to 8.37 ± 0.33 pmol.mg protein^–1^.20 s^–1^ and 7.95 ± 0.34 pmol.mg protein^–1^.20 s^–1^ in the presence of 20 and 50 µM of biotin methyl ester, respectively.	Biotin methyl ester and α-lipoic amide having modified carboxylic acid group and do not effectively compete with the process of biotin transport by SMVT.	
			α-Lipoic amide	Biotin (5 µM) uptake slightly reduced from 9.80 ± 0.60 pmol.mg protein^–1^.20 s^–1^ (control group) to 9.10 ± 0.44 pmol.mg protein^–1^.20 s^–1^ in the presence of 50 µM of α-lipoic amide.		
2	Human-derived colon cell line, Caco-2 cells	[^3^H]biotin	Unlabelled biotin	The transport of [^3^H]biotin (4 nM) reduced from 1.69 ± 0.04 pmol.mg protein^–1^.3 min^–1^ to 0.664 ± 0.03 pmol.mg protein^–1^.3 min^–1^ and 0.56 ± 0.02 pmol.mg protein^–1^.3 min^–1^ in the presence of 25 and 50 µM of unlabelled biotin, respectively.	Co-treatment with unlabelled biotin and desthiobiotin led to inhibition of the transport of [^3^H]biotin. This study establishes the critical role of the carboxylic acid group in the biotin molecule for its transport by SMVT.	88
			Desthiobiotin	The transport of [^3^H]biotin (4 nM) reduced from 1.69 ± 0.04 pmol.mg protein^–1^.3 min^–1^ to 0.95 ± 0.02 pmol.mg protein^–1^.3 min^–1^ and 0.73 ± 0.01 pmol.mg protein^–1^.3 min^–1^ in the presence of 25 and 50 µM of desthiobiotin, respectively.		
			Biocytin	The uptake of [^3^H]biotin (4 nM) reduced from 1.69 ± 0.04 pmol.mg protein^–1^.3 min^–1^ to 1.47 ± 0.12 pmol.mg protein^–1^.3 min^–1^ and 1.16 ± 0.05 pmol.mg protein^–1^.3 min^–1^ in the presence of 25 and 50 µM of biocytin, respectively.	Biotin analogs like biocytin and biotin methyl ester having modified carboxylic acid group and had a non-significant effect on [^3^H]biotin transport by SMVT. While tetrahydroimidazolone ring (absent in diaminobiotin) fused with tetrahydrothiophene ring also seems to be important for interaction with SMVT.	
			Biotin methyl ester	25 and 50 µM concentrations of biotin methyl ester and diaminobiotin had a non-significant effect on [^3^H]biotin (4 nM) uptake.		
			Diaminobiotin			
3	Kidney cells (MDCK-MDR1)	[^3^H]biotin	Unlabelled biotin	Compared to the control, the uptake of [^3^H]biotin (10 nM) significantly reduced to 9.03 ± 1.51%, 51.50 ± 7.46%, and 16.27 ± 0.47% in the presence of 1 mM, 10 µM, and 100 µM of unlabelled biotin, respectively.	Data analysis showed that the unlabelled biotin, pantothenic acid, lipoic acid, valeric acid, and desthiobiotin significantly inhibited the uptake of [^3^H]biotin in the presence of varying concentrations. While biotin analogs with a modified carboxylic acid such as biotin methyl ester and biocytin do not effectively compete with the uptake of [^3^H]biotin.	33
			Pantothenic acid	Compared to the control, the uptake of [^3^H]biotin (10 nM) significantly reduced to 9.12 ± 1.39%, 27.22 ± 1.06%, and 25.62 ± 2.06% in the presence of 1 mM, 10 µM, and 100 µM of pantothenic acid, respectively.		
			Desthiobiotin	Compared to the control, the uptake of [^3^H]biotin (10 nM) significantly reduced to 15.61 ± 2.41%, 40.97 ± 4.29%, and 16.67 ± 1.69% in the presence of 1 mM, 10 µM, and 100 µM of desthiobiotin, respectively.		
			α-Lipoic acid	Compared to the control, the uptake of [^3^H]biotin significantly reduced to 6.97 ± 0.82% in the presence of 1 mM of α-lipoic acid.		
			Valeric acid	The uptake of [^3^H]biotin was significantly reduced to 60.39 ± 4.02% in the presence of 1 mM of valeric acid, compared to the control.		
4	Human-derived retinoblastoma cell line (Y-79)	[^3^H]biotin	Unlabelled biotin	The uptake of [^3^H]biotin (10 nM) significantly decreased to 45.2% and 73.6% in the presence of 50 µM and 100 µM of unlabelled biotin, respectively when compared to the control group.	Competition with unlabelled biotin, lipoic acid, pantothenic acid, desthiobiotin, and valeric acid significantly decreased the uptake of [^3^H]biotin at various concentrations. While the biotin analogs, such as biocytin and biotin methyl ester were non-significant to decrease the uptake of [^3^H]biotin.	93
			α-Lipoic acid	The uptake of [^3^H]biotin (10 nM) significantly decreased to 53.9% and 74.1% in the presence of 50 µM and 100 µM of lipoic acid, respectively, when compared to the control group.		
			Pantothenic acid	The uptake of [^3^H]biotin (10 nM) significantly decreased to 39.2% and 65.6% in the presence of 50 µM and 100 µM of pantothenic acid, respectively as compared to the control group.		
			Desthiobiotin	Compared to the control group, the uptake of [^3^H]biotin (10 nM) significantly decreased to ∼35% and ∼65% at 50 and 100 µM of desthiobiotin, respectively.		
			Valeric acid	Compared to the control group, the uptake of [^3^H]biotin (10 nM) significantly decreased to 38.6% at 100 µM of valeric acid.		
5	Breast cancer cells (T47D)	[^3^H] biotin	Unlabelled biotin	Compared to the control group, the uptake of [^3^H]biotin (8.3 nM) significantly reduced to 49.24 ± 3.48%, 22.41 ± 3.31%, and 20.74 ± 2.17% in the presence of 10, 50, and 100 µM of unlabelled biotin, respectively.	This study corroborates the strong competitive effect of unlabelled biotin, desthiobiotin, pantothenic acid, and lipoic acid on the uptake of 8.3 nM [^3^H]biotin in a concentration-dependent manner.	89
			Desthiobiotin	Compared to the control group, the uptake of [^3^H]biotin (8.3 nM) significantly reduced to 62.44 ± 2.25%, 36.1 ± 3.59%, and 22.33 ± 1.05% in the presence of 10, 50, and 100 µM of desthiobiotin, respectively.		
			Biocytin	Compared to the control group, the uptake of [^3^H]biotin (8.3 nM) decreased to 90.71 ± 11.70%, 79.25 ± 24.16%, and 76.56 ± 21.99% in the presence of 10, 50, and 100 µM of biocytin, respectively.	Biocytin produced a weak inhibitory effect on the uptake of [^3^H]biotin.	
			NHS–biotin	Compared to the control group, the uptake of [^3^H]biotin (8.3 nM) decreased to 60.75 ± 4.45%, 38.41 ± 10.71%, and 28.17 ± 10.77% at 10, 50, and 100 µM, respectively.	As NHS–biotin is a reactive compound, there is a high probability that it could acylate the cell surface proteins or get hydrolysed in the culture medium under experimental conditions ^115^. In such a case, the hydrolysed product would effectively compete with the [^3^H]biotin for uptake by SMVT.	

An extensive study by Finn and coworkers^119^ demonstrates the high specificity of SMVT towards modification in the structure of pantothenic acid (Figure 1). In the study, pantothenic acid derivatives were tested for competitive reduction of [^3^H]biotin uptake by SMVT in human embryonic kidney (HEK) cells transfected with human SMVT using LC–MS/MS. The SAR analysis (totally 18 derivatives) showed that modification of the carboxylic acid to an amide, ester, alcohol, or triazole of the β-alanine fragment of the pantothenic acid resulted in a significant loss of biotin uptake inhibitory activity. Further, it was found that only pantothenic acid derivatives with a free carboxyl group were tolerated by the transporter as shown by inhibition of biotin uptake and sodium-dependent transport of these derivatives. Overall, this study shows the high specificity of SMVT towards the modifications in the pantothenic acid structure and delineates the importance of the free carboxylic acid group of pantothenic acid for SMVT-mediated transport. Such results further corroborate the data from biotin uptake studies discussed in the previous SAR section, which emphasises the need for a free carboxylic acid group for SMVT-mediated transport.

**Figure 3.**
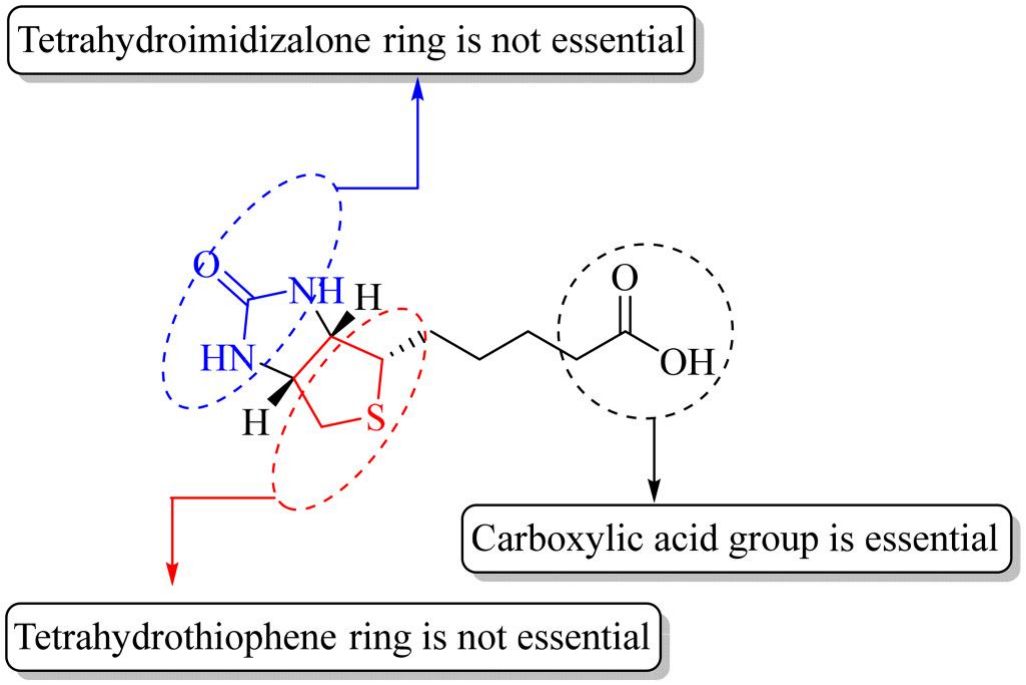
A general representation of SAR of SMVT substrates.

All the studies point to one thing: the presence of a carboxylic acid group is essential^72^. If one examines the mechanism through which SMVT transports a substrate, the requirement for a carboxylic acid is consistent with the driving force being electrochemical via a sodium ion gradient (Figure 2). In this regard, Table 1 and Figure 3 provide a summary of results from various competition experiments to show the structural properties of biotin crucial for its recognition by SMVT.

One of the central questions for this review is the vast body of literature examples of various drug–biotin conjugates synthesised through amidation or esterification of the biotin carboxylic acid group^8^^,^^31^^,^^45^^,^^120^^,^^121^. Despite the requirement of a free carboxyl group for SMVT-mediated transport and the lack of a carboxyl group in commonly used biotin–drug conjugates, most studies attribute the targeted delivery of such conjugates due to binding to a “biotin receptor,” which is sometimes inaccurately used to mean biotin transporter. As discussed earlier, the concept of a “receptor” is different from that of a “transporter”. In the subsequent sections, we provide a brief description of such examples and analyse the apparent inconsistency between the structural need for SMVT transport and the key features of such biotin conjugates.

## Biotin conjugates in delivery applications

**Figure 4.**
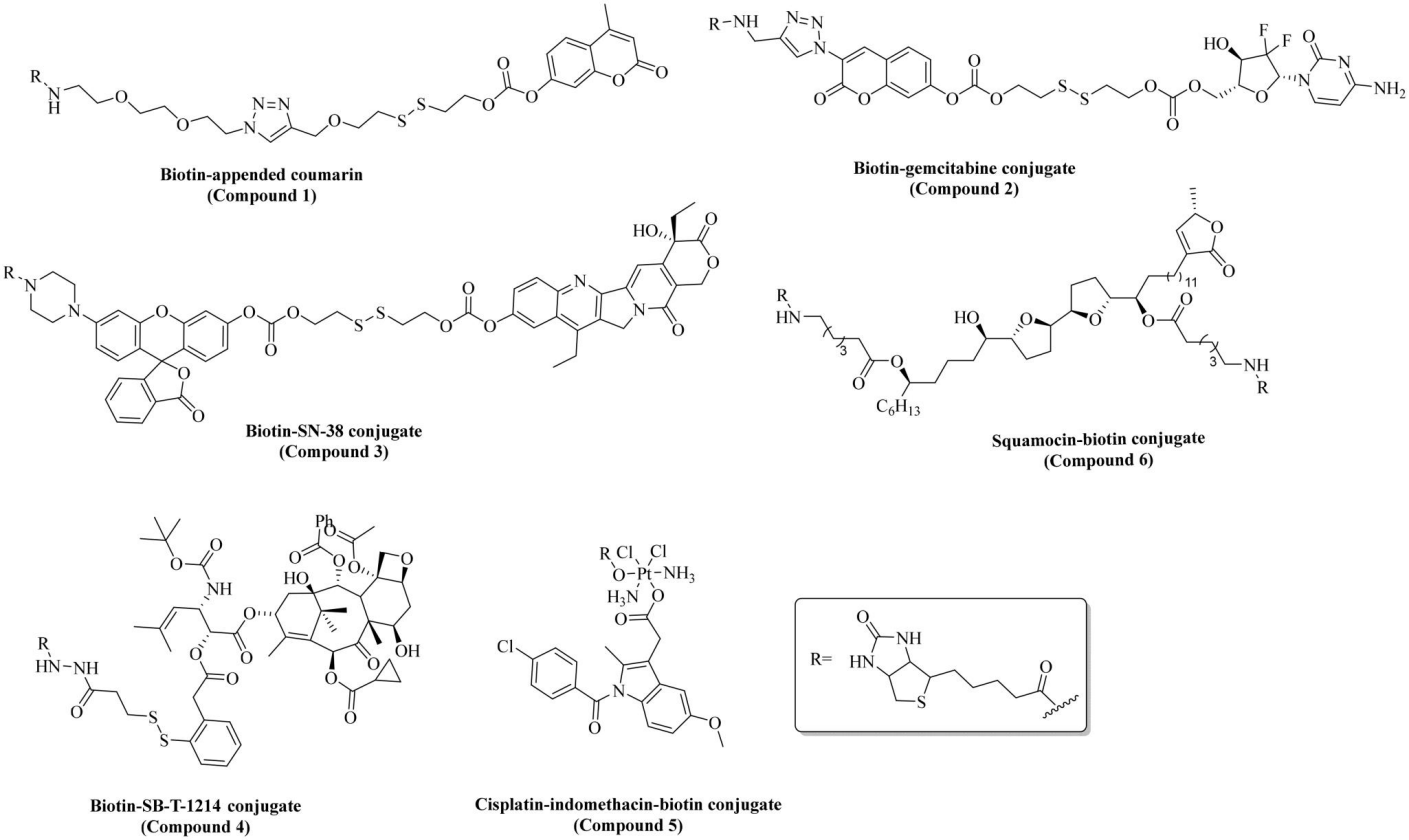
Chemical structures of the biotin-conjugates described in Table 2.

Biotin has been extensively investigated for various applications including delivery of both therapeutic and imaging agents^31^^,^^45^^,^^69^^,^^78^^,^^86^^,^^122^. Without being comprehensive, we herein attempt to succinctly show the extent of applications of biotin conjugates. Table 2 displays examples of various types of applications of biotin conjugates. Mostly, biotin has been used as a vector to deliver a payload to cancer cells^31^^,^^86^. For instance, Kim and coworkers published their work on the development of a biotin-conjugated theranostic agent (compound **2**) containing gemcitabine (an anticancer drug) and coumarin (a fluorescent reporter) (entry 2, Table 2)^123^. As proposed by the authors, this multifunctional compound undergoes thiol-triggered release of gemcitabine and biotin-conjugated coumarin under physiological conditions. Briefly, compound **2** (10 µM) was incubated for 15 min with A549 cells (human lung carcinoma epithelial cells) and WI38 cells (Caucasian fibroblast-like foetal lung cells). Markedly, higher fluorescence was observed in A549 cells compared to the WI38 cells. Further, compound **2** was tested for anticancer activity using a cell viability assay. In comparison to the control group (no treatment), compound **2** reduced the cell viability by >80% at 1 µM, while non-biotinylated gemcitabine-coumarin conjugate reduced the cell viability by ∼50%. In addition, gemcitabine alone (1 µM) treatment resulted in ∼30% reduction of the cell viability compared to the control group (untreated). Subsequently, Kim and coworkers also reported biotin–coumarin conjugate (compound **1**) for selective labelling of cancer cells (entry 1, Table 2)^121^. In this study, biotin-conjugated coumarin (5 µM) showed a high level (quantitative data not provided) of fluorescence in A549 cells compared to WI38 cells after 20 min of incubation. Additional cell imaging studies showed a relatively high level of fluorescence (quantitative data not provided) in A549 cells for the biotin-conjugated compound when compared to its non-biotinylated derivative after 20 min of incubation. Overall, results from both articles show the ability of biotin to selectively deliver compounds to cancer cells. Though the enhancement effects seem small, biotinylation of anticancer drugs indeed provides beneficial effects. Additionally, biotin conjugation has been also extensively exploited for enhancing cancer cell accumulation of anticancer compounds such as SN-38 (entry 3, Table 2), SB-T-1214 (entry 4, Table 2), cisplatin (entry 5, Table 2), curcumin (entry 8, Table 2), squamocin (entry 9, Table 2), and paclitaxel (entry 10, Table 2)^46^^,^^80^^,^^124^^,^^126–128^. Generally, such biotin conjugates are synthesised through amidation or esterification of the biotin carboxylic acid (Figure 4). As discussed in the previous section, such transformations are contraindicated for interaction with the SMVT based on SAR studies and beg the question as to whether transport of such biotin conjugates is through the SMVT. In the subsequent sections, we probe the consequences of modification of the carboxylic acid on the transport of biotin conjugates reported in the literature for various applications.

**Table 2.** Applications of biotin in the development of drugs and diagnostics.

Entry	Application	Model	Biotin conjugate	Results	Ref.
1	Cancer diagnosis	A549 cells, *in vitro*	Coumarin (compound **1**)	In cell imaging experiments, biotin-appended coumarin conjugate (5 µM) showed strong fluorescence within 20 min of incubation in the cells compared to the compound without biotin. Hence, compound **1** is said to show the potential to be used as cancer-selective imaging probe.	121
2	Cancer-targeting theranostic	A549 cells, *in vitro*	Gemcitabine (compound **2**)	In a cell viability assay, compared to the untreated group, biotin-conjugated gemcitabine (1 µM) reduced the cell viability to <20% while the cell viability was found to be slightly >70% when treated with gemcitabine (1 µM) alone for 72 h.	123
3	Cancer-targeting theranostic	HeLa cells, *in vitro*	SN-38 (compound **3**)	At 10 µM, the cell viability after treatment SN-38 (alone) and biotin-conjugated SN-38 were found to be ∼20% and ∼40% after 48 h incubation, respectively.	80
4	Tumour-targeting drug delivery	L1210F cells, *in vitro*	Paclitaxel derivative SB-T-1214 (compound **4**)	Biotin-conjugated SB-T-1214 resulted in a marginal reduction of IC_50_ value (8.8 nM) compared to the non-conjugated compound (9.5 nM) after 72 h incubation as shown by the MTT assay.	46
5	Cancer-targeted drug delivery	PC-3, SGC7901, SGC7901/CDDP cells, *in vitro*	Cisplatin-indomethacin hybrid (compound **5**)	1–3-fold improved cellular accumulation demonstrated by the biotin conjugate over the parent drug alone as determined by the measurement of the intracellular cisplatin content using ICP-MS after 12 h of incubation.	124
6	Drug delivery	Gene-knocked out diabetic (SLAC/GK) rats, *in vivo*	Insulin	Compared to the original blood glucose concentration, the maximum reduction of blood glucose level was found to be 56% and 70% after 20 IU/kg dose administration of biotinylated and non-biotinylated liposomes, respectively. Such result is considered proof of concept that biotinylated liposomes can be employed as carriers for oral drug delivery.	125
7	Drug delivery	Caco-2 cells, *in vitro*	Salmon calcitonin	A 2.5- and 4-fold increase in apical-to-basolateral permeability was demonstrated by mono- and di-biotinylated conjugates, respectively, as opposed to the non-biotin conjugated form. Similar to the entry 6, this study is said to show the potential of biotinylated carriers for improving oral drug delivery.	83
8	Cancer-targeted drug delivery	MDA-MB-436 cells, *in vitro*	Curcumin contained in poly (ethylene glycol)–poly (epsilon-caprolactone) micelles	The cellular uptake of biotinylated micelles showed a 4-fold improvement over its non-biotinylated micelles, as shown by their mean fluorescence density values after 2 h incubation.	126
9	Cancer-targeted drug delivery	P815 cells, *in vitro*	Squamocin (compound **6**)	Biotin-conjugated squamocin resulted in the reduction of IC_50_ value (0.75 ± 0.02 µM) compared to the squamocin alone (19.80 ± 1.18 µM) after 72 h incubation as shown by the MTT assay.	127
10	Cancer-targeting theranostic	HeLa cells, *in vitro*	Paclitaxel surface-functionalised on gold nanoparticle (AuNP)	Biotin conjugate AuNP showed >2-fold improvement in cytotoxicity in comparison to its non-biotin conjugated version after 24 h incubation.	128

## Activated biotin for cell-surface protein labelling work

Relevant to the discussion of biotin-mediated drug delivery issues, it is important to address another seemingly contradictory application of biotin: selective covalent labelling of (only) cell-surface proteins using biotinylated reactive agents^129–136^. Such experiments utilise the high acylating reactivity of *N*-hydroxysuccinimide–biotin conjugates (NHS–biotin) and sulfo-NHS-SS-biotin (Figure 1) to specifically conjugate and subsequently extract cell-surface proteins using streptavidin beads^130^^,^^131^^,^^134^^,^^137^. Such a design is predicated on two characteristics of biotin: low passive permeability across the cell membrane (and thus only labels cell surface proteins) and high affinity for streptavidin (and avidin)^122^, which are bacterial proteins with high affinity for biotin and are often used in isolation of biotinylated conjugates. Furthermore, such protein labelling work is not limited to cells without biotin transporter(s). A natural question is whether such protein labelling experiments would contradict the idea of transporter-mediated uptake of biotin conjugates. The answer seems to be with the temperature-dependent nature of the biotin transport^33^^,^^46^^,^^79^^,^^89^^,^^93^. Generally, cell-surface labelling experimental protocols apply low temperature (generally performed at 4 °C) during the incubation of biotinylating reagents with cells to minimise uptake and afford selectivity for surface proteins^129^^,^^133^^,^^138^. However, there are also examples of labelling experiments conducted at room temperature or 37 °C^139–142^. It is not clear whether the success of such experiments is correlated with low or lack of expression of the biotin uptake system. At this point, this issue needs to be examined to understand the mechanistic implications. It is possible that the acylating reaction is kinetically faster than transport, leading to the selective labelling of cell surface proteins. Another possibility is for NHS–biotin to acylate any biotin transporter present, leading to covalent inhibition. All these also suggest the possibility of using NHS–biotin or similarly activated biotin-based acylating agent for identifying any unknown “biotin receptor”.

## Intriguing possibilities

With all the reports of targeted drug delivery by using biotin conjugates, there are very important unresolved issues. First, most of the SAR studies in biotin transporter binding uniformly specify the need for a carboxyl group. This is described in detail in section “The structure–activity relationship: what might the SAR imply in terms of uptake mechanism(s)?” (Figure 3 and Table 1). However, with all the biotin conjugates, the carboxyl group is used for conjugation through the formation of an amide (or occasionally an ester) bond. This means the elimination of the free carboxyl group. Then, there is the question as to why a conjugate with a modified carboxyl group binds to a transporter that has the structural requirement of a free carboxyl group in its substrate. Do these biotin conjugates really rely on the biotin transporter(s) for targeting and delivery of a payload? If not, what is/are the molecular target(s) for these biotin conjugates? Second, in the literature, the term “biotin receptor” is often used^79–81^^,^^83^^,^^85^^,^^86^. However, there has not been a “biotin receptor” reported^72^^,^^73^^,^^75^^,^^77^^,^^91^^,^^92^^,^^109^^,^^118^. Here, there is the need to emphasise that “transporter” and “receptor” are two different concepts in biology. Again, structurally “transporters mediate the passage of molecules across cell membranes by alternating between inward- and outward-facing states, while receptors undergo intracellular structural rearrangements that initiate signaling cascades^87^”. There will be a need to conduct extensive studies of this issue. If biotin-based drug delivery does not rely on a biotin transporter (or specifically SMVT), then why is the reported correlation of biotin transporter expression level with the ability for biotin conjugation-based targeting? Third, the biotin transporters tend to have *K*_m_ in the low to mid-micromolar range^88^^,^^90^^,^^92^^,^^102^^,^^143^. Then why some biotin conjugates in the nanomolar range are effective in targeted delivery? Lastly, SMVT has a molecular weight of 68.6 kDa and transports small molecules such as biotin, α-lipoic acid, pantothenic acid, and valproic acid effectively^70^^,^^102^. However, there have been reports of biotin-mediated transport of payloads of high molecular weight and/or size such as biotin-conjugated polypeptides, synthetic polymers, and nanoparticles^31^^,^^84^^,^^103^^,^^144^. Molecular size plays a vital role in determining its cellular uptake mechanism. For example, nanoparticles tend to undergo an endocytic uptake mechanism due to their large size while small molecules may enter cells passively or via active transport depending on their physiochemical properties^145–147^. In the following subsections, we pose these questions and present our analysis.

### Transporter vs. receptor

Again, earlier SAR studies have uniformly shown the critical role of the carboxylic acid group for SMVT-mediated uptake of biotin. However, biotin conjugates in targeted delivery studies uniformly lack this functional group. For instance, Chen et al.^103^ developed biotinylated chitosan surface-modified poly(d,l-lactide-*co*-glycolide) nanoparticles (Bio-PLGA NPs) to deliver epirubicin (anticancer agent) to MCF-7 cells *in vitro*. Compared to epirubicin-loaded PLGA (54.5%), cell viability was somewhat lower (36.1%) when treated with Bio-PLGA NPs for 120 h. The cytotoxic effect exerted by Bio-PLGA NPs was attributed to the enhanced accumulation of the drug inside cancer cells. Cellular internalisation of NPs was investigated by measuring epirubicin fluorescence signals using flow cytometer. In MCF-7 cells, a ∼41% increase in fluorescence was observed after treatment with Bio-PLGA NPs for 2 h in comparison to epirubicin-loaded PLGA. Furthermore, experiments were carried out to understand the mechanism of the cellular accumulation of Bio-PLGA NPs. In this regard, cells were pre-treated with 10 µg/mL (40.93 µM) of biotin in serum-free media at 37 °C. After 24 h, cells were washed and treated with Bio-PLGA NPs at epirubicin concentration of 10 µg/mL (18.4 µM) for 2 h and further analysed by flow cytometry. Pre-treatment with biotin reduced the uptake of Bio-PLGA NPs to ∼60% of the control group. On the surface, the results seem to be consistent with biotin transporter-mediated accumulation. Further, endocytic inhibitors chlorpromazine, filipin, and amiloride were used to probe the mechanism of receptor-mediated endocytosis. In all the endocytosis experiments, endocytic inhibitors were incubated with MCF-7 cells for 24 h at 37 °C prior to the incubation with NPs for 2 h before flow cytometry analysis. Chlorpromazine is commonly used to inhibit clathrin-mediated endocytosis, which is widely regarded as a major pathway to transport cargo through endocytosis^148–151^. Amiloride acts as an inhibitor of micropinocytosis by inhibiting Na^+^/H^+^ exchange proteins on the cell membrane^152^. In addition, caveolae-mediated endocytosis can be inhibited by filipin^153^. These pathways have been classified as fundamental routes for intracellular trafficking of various extracellular molecules including nutrients, hormones, and nanoparticle^149^^,^^154^^,^^155^. By measuring epirubicin fluorescence using flow cytometry, a >50% reduction of cellular internalisation of Bio-PLGA NPs was noted after pre-treatment with chlorpromazine (10 µg/mL) compared to the control group. About 15% and 30% reduction of Bio-PLGA NPs cellular uptake were noted after pre-treatment with filipin (5 µg/mL) and amiloride (10 µg/mL), respectively, when compared to the respective control groups. The inhibitory effect of amiloride on biotin transport is well-known as the blockade of the Na^+^ channel, suggesting a possibility of impairment of sodium-dependent transport of biotin by SMVT^117^. Interestingly, results also suggest a receptor-mediated process, since SMVT transporter does not require endocytosis for the translocation of biotin into the cells. Endocytosis is part of the receptor-mediated trafficking of cargo^156^^,^^157^. However, the identity of this “receptor” is not known and is probably not the biotin transporter. In contrast, a study by Mitra and coworkers reported that the intracellular uptake of [^3^H]biotin remains unaltered in human-derived prostate cancer cells (PC-3) after pre-treatment with colchicine (100 µM) compared to the control group^117^. Noteworthy, colchicine inhibits endocytosis by disrupting microtubule assembly^109^. Sinko and coworkers^158^ also presented evidence against endocytosis^103^ being the uptake mechanism. Briefly, enhanced accumulation of biotinylated polyethylene glycol (PEG)-based Tat9 peptide (PEG:(R.I-Cys-K(biotin)-Tat9)_8_) (29 kDa) was observed in Chinese hamster ovary (CHO) cells transfected with human SMVT (CHO/hSMVT). In the competition experiments, biotin-conjugated peptide (0.1 µM) was co-incubated with 50 µM of various compounds. After 10 min coincubation, free biotin diminished the uptake of biotin-conjugated peptide to ∼35%; desthiobiotin and biocytin inhibited the uptake to ∼45% as compared to the control group. Further, pantothenate, PEG–biotin (PEG MW = 3400), and biotin–PEG–biotin (PEG polymer containing two biotin molecules) reduced the uptake of the biotin-conjugated peptide to ∼55–60% of the control group. Such results show competitive inhibition of biotin-conjugated peptides by molecules with a free carboxyl group (biotin, desthiobiotin, and pantothenate) as well as molecules with an amide group (biocytin, PEG–biotin, and PEG–biotin–biotin). Overall, results from these studies further emphasise the need to carefully look into the molecular mechanism(s) of biotin transport. Along this line, Russell-Jones et al. published a comprehensive study investigating the participation of an SMVT-independent uptake system in the absorption of biotin-conjugated rhodamine-labelled hydroxypropyl-methacrylamide (Bio-Rh-HPMA; 22 kDa) and biotin-conjugated quantum dots (B-Qdots; 10–12 nm)^116^. Bio-Rh-HPMA was synthesised through conjugation using biotin’s carboxylic group. Further, different tumour cells such as ID8, RENCA, MMT, and Ov2008 were chosen to study the *in vitro* uptake mechanism of Bio-Rh-HPMA (25 µL of 20 mg/mL) using fluorescent microscopy. After 4 h incubation, histological examination showed punctate staining of the tumour cells due to the accumulation of the fluorescent Bio-Rh-HPMA in vesicle-like structures, implying endosomal uptake of the Bio-Rh-HPMA. Further, experiments were also carried out *in vitro* with B-Qdots. RD995 cells were incubated with 2.5 µL B-Qdots in 500 µL culture medium and monitored at 1 h and 5 h time points. The concentration B-Qdots was not provided in the article. However according to the catalogue (catalogue number: Q10321MP) from the manufacturer (Invitrogen, Carlsbad, CA), it should be 2 µM (stock solution). Hence, cells were incubated with 10 nM B-Qdots. A time course experiment using a fluorescent microscope showed clustering of the fluorescent B-Qdots on the cell surface at 1 h, with minimal internalisation. B-Qdots in subcellular vesicles were observed after 5 h. The phenomenon of surface-clustering and endosomal uptake are the characteristics of receptor-mediated uptake of cargo^156^^,^^157^^,^^159–161^. Such results are not strongly consistent with polymer uptake by SMVT. Furthermore, the uptake of Bio-Rh-HPMA (100 µL of 20 mg/mL for 6 h) in M109 metastatic tumour-bearing mice was also studied. Imaging studies showed that Bio-Rh-HPMA was predominantly absorbed by the tumour cells growing immediately adjacent to normal intestinal tissue. As normal intestinal cells also express SMVT^32^, the selective uptake of fluorescent molecules by tumour cells suggests the possibility of an SMVT-independent uptake system responsible for the absorption of Bio-Rh-HPMA in tumour cells. Clearly, analysis of these studies raises several questions that warrant further investigations to delineate the cellular uptake mechanism of biotin and its conjugates.

Adding to the perplexity, a study by Smirnov and coworkers^94^ revealed unique results in a competition assay between biotin and biotin-conjugate with PEG through an amide bond. In this study, attachment of HeLa cells onto the flat glass surface was examined. The glass surface was decorated with 1% biotin through a PEG linker. For the competition assay, the cell suspension was mixed with 0.8 mM biotin. Within 1 min, the cells were further incubated for 15 min with modified glass slides to probe the attachment of the cells on the surface. Unlike the reduction of biotin-conjugate uptake in the presence of biotin as reported by Chen et al.^103^ and other researchers^46^^,^^63^^,^^162–164^, a 3–4-fold enhancement in attachment of cells onto the modified glass surface was recorded when compared to experiments without added biotin. Similarly, an augmentation of attachment of cells onto the modified glass surface was also observed with MCF-7 cells in the competition experiment. Incidentally, a study by Wei and coworkers reported that excess biotin (4 mM) did not impair the uptake of biotinylated Atto565 (2 µM) in a competition assay in *E. coli*^95^. In this study, the authors investigated the impact of biotin conjugation on the accumulation of Atto565 (a commercially available fluorescent dye) in *E. coli*. Interestingly, fluorescence measurement of the biotin-conjugated Atto565 showed ∼2-fold higher accumulation of Atto565 upon biotinylation. Once again, such unexpected results highlight the differential mechanism involved in the interaction of biotin and biotin-derivatised molecules with their specific uptake system(s). Given the wide-spread interest in using biotin conjugates in targeted drug delivery, these results suggest the need for further mechanistic studies to probe the cellular uptake of biotin conjugates.

### The Michaelis–Menten constant

Similar to enzymes, SMVT also displays Michaelis–Menten saturation kinetics to transfer substrates across the cell membrane^33^^,^^113^^,^^158^. Using this Michaelis–Menten equation, several researchers have extensively studied SMVT-mediated uptake of biotin. Previously, the *K*_m_ value of biotin was calculated to be 9.5 µM in Caco-2 cells^88^, 32.52 µM in rabbit corneal epithelial cells, 63.8 µM in Statens SerumInstitut Rabbit Cornea (SIRC) cells^90^, and 19.7 µM in human colonic epithelial NCM460 cells^92^. Along this direction, Sinko and coworkers have investigated the uptake of a biotin conjugate, i.e. [^3^H]PEG-biotin (PEG MW = 3400) in Caco-2 cells^158^. The kinetic studies showed significant inhibition of [^3^H]PEG-biotin uptake by free biotin with an inhibition constant (*K*_i_) of 6.78 µM. The *K*_i_ for desthiobiotin, biocytin, and biotin–PEG–biotin was calculated to be 11.47, 14.01, and 19.08 µM, respectively. The *K*_m_ value of biotin–PEG was calculated to be 6.61 µM in Caco-2 cells. Once again, these results clearly show competitive inhibition of biotin–PEG by biotin and its derivatives without a free carboxylic acid. Noteworthy, biocytin has been reported to be a weak competitor of biotin transport by SMVT in T47D cells (entry 5, Table 1)^89^, Caco-2^88^, and other cell lines^89^^,^^118^. The *K*_m_ value of biotin–PEG was also found to be similar as shown by biotin towards SMVT in Caco-2 cells^88^. Based on the *K*_m_ values, authors stated that both biotin and its conjugates devoid of a carboxylic acid group share the same uptake mechanism. Simultaneously, such results also contradict earlier findings, which indicate the essential nature of a free carboxylic acid group for SMVT-mediated transport. Hence, this example further emphasises the need to carefully examine the molecular mechanism(s) of biotin transport.

### Small vs. large molecule

In the past two decades, biotin has been widely used as a targeting ligand for delivering small molecules such as doxorubicin, gemcitabine, and large molecules such as polypeptides, polysaccharides, nanoparticles, and micelles^31^^,^^45^^,^^116^^,^^162^^,^^165^. If we take a look at the molecular weights of the currently known SMVT substrates, all of these molecules are small molecules (Figure 1). Of note, the molecular weight of SMVT is calculated to be 68.6 kDa^70^^,^^102^. Generally, molecules <1 kDa molecular weight are categorised as small molecules^145^^,^^147^. However, it is imperative to ask the question of whether biotin-conjugated small molecules share the same uptake mechanism as conjugated macromolecules or particles. For instance, Chen et al. applied Bio-PLGA NPs (10–30 kDa) to selectively deliver epirubicin to cancer cells^103^. These nanoparticles have 50–150 times higher molecular weights than biotin. Generally speaking, there is the question of whether a transporter meant for a small molecule is capable of transporting a nanoparticle. As described in the previous section, Bio-PLGA NPs undergo the endocytosis pathway to enter cells as demonstrated by the experiments applying endocytosis inhibitors, viz., chlorpromazine, filipin, and amiloride. Similarly, experiments conducted by Russell-Jones et al. employing Bio-Rh-HPMA (22 kDa) also indicate the participation of endocytic pathways to deliver Bio-Rh-HPMA to tumour cells as demonstrated by imaging studies^116^. Therefore, these findings point to the possibility of the presence of multiple mechanisms for the uptake of such biotin-conjugates that are devoid of a carboxylate group and comprise high molecular weight. Endocytosis for the trafficking of biotinylated cargos seems to be a distinct possibility as shown by the experiments applying endocytosis inhibitors.

## Conclusions

Biotin has been widely studied as a vector for targeted drug delivery. SMVT has been recognised as the major transporter of biotin. Based on SAR studies, it has been found that the free aliphatic carboxylic acid of biotin plays a vital role in its recognition by SMVT. Importantly, the modification of the carboxylic acid of biotin to an amide or ester functional group seems to abolish its SMVT-mediated uptake. However, biotin-mediated drug delivery relies on the payload moiety being conjugated through amidation or esterification of the free carboxylic acid of biotin. Despite being devoid of free carboxylic acid, these biotin conjugates are reported to target biotin uptake system(s) for enhanced cell permeation. The results from the competition assay of biotin conjugates with free biotin and endocytosis experiments imply the involvement of multiple mechanisms for the uptake of biotin conjugates. In this regard, further studies are warranted to delineate the cellular uptake mechanism of biotin conjugates. A clear understanding of such mechanism(s) will be critical for the future design and development of biotin-based therapeutics.
